# Spatial Overlap of Grey Seals and Fisheries in Irish Waters, Some New Insights Using Telemetry Technology and VMS

**DOI:** 10.1371/journal.pone.0160564

**Published:** 2016-09-28

**Authors:** M. Cronin, H. Gerritsen, D. Reid, M. Jessopp

**Affiliations:** 1 MaREI Centre, Beaufort Building, Environmental Research Institute, University College Cork, Ringaskiddy, Co. Cork, Ireland; 2 Marine Institute, Oranmore, Co. Galway, Ireland; Hawaii Pacific University, UNITED STATES

## Abstract

Seals and humans often target the same food resource, leading to competition. This is of mounting concern with fish stocks in global decline. Grey seals were tracked from southeast Ireland, an area of mixed demersal and pelagic fisheries, and overlap with fisheries on the Celtic Shelf and Irish Sea was assessed. Overall, there was low overlap between the tagged seals and fisheries. However, when we separate active (e.g. trawls) and passive gear (e.g. nets, lines) fisheries, a different picture emerged. Overlap with active fisheries was no different from that expected under a random distribution, but overlap with passive fisheries was significantly higher. This suggests that grey seals may be targeting the same areas as passive fisheries and/or specifically targeting passive gear. There was variation in foraging areas between individual seals suggesting habitat partitioning to reduce intra-specific competition or potential individual specialisation in foraging behaviour. Our findings support other recent assertions that seal/fisheries interactions in Irish waters are an issue in inshore passive fisheries, most likely at the operational and individual level. This suggests that seal population management measures would be unjustifiable, and mitigation is best focused on minimizing interactions at nets.

## Introduction

Where their ranges overlap, humans compete globally with top marine predators such as marine mammals for resources, including food. A number of studies in the North Atlantic have examined competition between grey seals and fisheries [1–3]. In the North-Sea and on the Scotian Shelf, grey seals have been associated with the failure of cod stocks to recover on both sides of the North East Atlantic [3, 4]. Indeed the problem of seal fishery interactions extends to the Baltic and Mediterranean Seas [5, 6] and the South Atlantic and Pacific Oceans [7–9]. Concerns have been expressed by the fishing industry in Ireland about the impact of seals on fish stocks [10], however our ability to quantify wide-scale interactions is often hampered by lack of robust data. Estimating the impact of a top predator such as seals, on a fishery in terms of biomass removal requires detailed knowledge of predator population size and distribution, energy requirements, diet composition as well as information on the energy content of prey. Studies to date suggest seal diet varies spatially and temporally [11, 12], and caution needs to be exercised in using data from a limited number of study sites to extrapolate to a wide area/population level. In the relatively data-rich area of southwest Ireland where recent research efforts have produced robust data on seal diet, Houle *et al*. [13] investigated the impact of local seal populations on fisheries using a size and trait-based marine community model, and suggest that the impact of seals on fisheries in the region are minimal compared to the amount of fish taken annually by the fishery. They concluded that seals are not likely to be competing directly with the fisheries in southwest Ireland. Other approaches using tracking technologies investigated the spatial overlap in grey seal foraging effort and human fishing effort off the west coast [14]. This study demonstrated a significantly low spatial overlap between tagged grey seals and the offshore whitefish fishery on the Irish continental shelf. These studies were primarily focused on the whitefish demersal fisheries off the west coast, which although a significant portion of fisheries in Irish waters, are only one sector in a European context. Unsurprisingly, the findings are contested by the fishing industry in Ireland, who believe that the results do not reflect the larger issue. Recent research conducted on ‘passive fisheries’ in Irish waters, i.e. fisheries using static gear e.g. tangle, trammel and gill netters, as opposed to active gear like trawls, suggests that these are the fisheries competing most with seals in Irish waters, with up to 50% loss attributed to seal depredation of catches [15]. This and other studies [10] suggests that seal/fisheries interactions are indeed a significant issue in passive fisheries in Irish waters, where seals are damaging and/or removing catch from static nets, particularly in gillnet fisheries targeting Pollack and hake and tangle net fisheries targeting monkfish and turbot. Such ‘operational interactions’ also potentially pose a conservation threat to seals, with high seal by-catch recorded in large mesh tangle net fisheries off the west coast [16].

Previous efforts looking at the spatial overlap of tagged seals with fishing vessels excluded this ‘passive fishery’ sector, as most of these vessels are <15m, and prior to 2012 positional data under the Vessel Monitoring System (VMS) was not available for vessels of this size. The system, a legal requirement under EC Regulation 2244/2003, was extended in 2012 to include vessels 12m-15m. Using an extended sample of tagged seals in an area with a wider representation of the Irish fishing fleet and relatively large colonies of grey seals, we examine the overlap of seals with active and passive fisheries in the Celtic and Irish Seas. Based on levels of depredation experienced in passive fisheries, we tested the hypothesis that there would be significant spatial overlap between seal foraging and passive fisheries.

## Methods

### Seal capture and tag deployment

Capture of grey seals and deployment of Fastloc/GSM tags (SMRU Ltd, UK) was carried out at haul-out sites on the Raven Point, Wexford Harbour Co. Wexford, southeast Ireland (52°13'53N 6°19'23W) in March 2013 and 2014. Grey seals use the Raven Point sand spit as a moult site between December-April each year and grey seals use of the area is relatively high (>100 seals) all year round. Up to 780 seals have been recorded at the site in July 2013 [17]. The tags were glued to the animals’ fur and therefore tagging was conducted in April to coincide with the completion of annual moult and to maximise the period of tag attachment. Seals were captured at the haul-out site using a combination of techniques including a seine net deployed at speed from a rigid inflatable boat directly in front of the site, and in hoop nets on land [14]. The captured seals were restrained in hoop nets throughout the administration of the anaesthetic and prior to the tagging procedure. Seals were weighed to the nearest kg and anaesthetised using 0.05ml of Zoletil (Virbac; a combination of a dissociative anesthetic agent, tiletamine hypochloride, and a tranquilizer, zolazepam hypochloride) per 10kg delivered intravenously. Males were approximately 20% ‘under-drugged’ due to risk of entering deep dive reflex while under anaesthetic. The fur was dried with paper towels and degreased using acetone and the tag was secured in place using either fast setting epoxy resin (RS components) in 2013 or superglue (Loctite) in 2014 at the base of the skull. All seal handling and tagging procedures were approved by the University Ethics Committee (UEC) of University College Cork, and conducted under licence by NPWS License No C04/C023/2013, and C016/2014, and Irish Health Products Regulatory Authority Project Licence AE19130/P004. The tags (10x7x4cm,370g) incorporate Fastloc GPS (Wildtrack Telemetry Systems, Leeds) and were programmed to attempt a location fix every 30 minutes but will only successfully do so if this coincides with the animal being at the surface. The tags use GSM technology to relay data ashore via a data link call, once within range of the coastal GSM zone [18].

### Estimating fishing effort of humans and seals

Since 1 January 2012, all fishing vessels in European waters exceeding 12 m in overall length have been required to transmit their position at least every 2 hours using a Vessel Monitoring System (VMS) (EC, 2003). While vessels were not individually monitored during this study, instantaneous vessel speed can provide a high level of vessel behaviour classification accuracy, with fishing operations particularly well identified [19]. Vessel behavior was characterized from VMS data based on gear-specific travelling speeds as a) inactive–vessel below minimum fishing speed (generally 0.1 knots for passive gears and 0.5 knots for active gears); b) fishing–vessel between minimum and maximum fishing speeds for its gear type; c) steaming–vessel exceeding maximum fishing speed for its gear type (generally 4.5 knots for passive gears and 5.5 knots for active gears). Vessel locations identified as either inactive or steaming were excluded from the analysis. The resulting VMS records represent the locations of fishing effort. In the case of active fisheries, this will be towing nets/lines, while for passive fisheries, this will represent locations where vessels are deploying and recovering gear, or searching for net markers in the fished area which should be a reasonable representation of fishing effort within grid cells. Fishing effort during the period for which seal telemetry data was available (April-Dec 2013, 2014) was estimated as time-in-space aggregated on a grid of 0.02° latitude by 0.02° longitude which corresponds to approximately 3km^2^.

Grey seals are known to spend time in the water near haul-out sites. Locations within 1km of the haulout site were excluded from the analyses to avoid including periods of haulout or inflating the importance of areas around haulout sites for foraging. Foraging effort of seals (hours spent per grid cell, on the same grid as the human fishing effort data) was estimated using time between GPS fixes. Interpolated dive locations are transmitted with the GPS fixes, however, to avoid introducing additional location error to the dataset, we use GPS locations derived from a minimum of 5 satellites. Dive data showed that seals undertook regular dives to the benthos across the entire track, and there was no evidence for periods of rest at sea (taken as a period of >1 hour between successive dives). Each GPS location is therefore considered a foraging location, and we used time-in-area as a proxy for foraging effort [20]. Any records with time intervals of more than 12 hours were removed to avoid assigning a disproportionate amount of effort to records that follow a period of missing data. An aggregate map of effort for all of the tagged seals and fisheries was created as well as separate maps for each individual seal and corresponding fisheries within the foraging range of each seal. The analysis was carried out in R (v 2.8.1, R Core Development Team) and ArcGIS V 9.3.

### Spatial overlap of seals and fisheries

The extent of the study area was defined by the distribution of the sample of seals tagged in Irish waters. Therefore fisheries data was limited to that which fell within the minimum convex polygon of the tagged seal locations.

The Morisita Horn Index of overlap C_MH*f*_ was used to estimate spatial overlap of seals and fisheries, where p_*f*_ *=* proportion of fishing effort, and p_*s*_ *=* proportion of seal foraging effort in each grid cell.

$$C_{MHf}=\frac{2\sum p_{f}p_{s}}{\sum p^{2}{}_{f}+\sum p^{2}{}_{s}}$$

The metric is unbiased with respect to sample size and diversity since the numerator is rescaled by the summed inner products of p_*f*_ and p_*s*_ [14, 21]. Low values of C_MH*f*_ suggest low overlap. To deal with potential temporal autocorrelation in the seal location points, data were randomly sub-sampled prior to analysis. We examined for spatial auto-correlation using the ACF (Auto and Cross Covariance and Correlation Function) and selected the lag at which the correlation is not statistically significantly different from 0. A block randomisation was performed where spatially independent blocks of the seal effort were randomised relative to the fishing effort. Significance levels for C_MHf_ were derived from 1000 permutations of the seal data i.e. the effort values per cell were permuted 1000 times, the fisheries overlap index calculated for each of these permutations, and C*data* compared to C*random* to examine the likelihood that the actual effort is distributed randomly. Indices of overlap were calculated for all seals and all fisheries across the entire study area. The fisheries data were then segregated into two categories; (i) active (including demersal and pelagic trawlers) and (ii) passive (gill nets, lines, trammel and tangle nets), and overlap between tagged seals and both active and passive fisheries examined. The analyses were also conducted on a sub-set of data limited to seal forage areas, using only 3km^2^ grid cells where seals were present, to closely examine what was happening within those areas, and on an individual seal basis.

## Results

### Tag deployment and operation

A total of 19 grey seals were captured and tagged on Raven Point, Co Wexford April 2013 (n = 9) and 2014 (n = 10). Six tags malfunctioned in 2013, and 2 tags were shed within days of attachment in 2014, resulting in a total of 11 seals successfully tracked. Weights of these 11 seals ranged from 71kg to 204kg (Table 1) and included 5 females and 6 males. Tags operated for approximately 3–4 months (mean duration 97 days; maximum 278 days). In total 1074 days of data were collected from the 11 grey seals with up to 12 location fixes per day per seal.

**Table 1:** Tagged seal statistics and tagging duration.

Seal	Gender	Weight/kg	Tag duration/days
**2**	M	192	42
**4**	F	118	163
**6**	M	95	37
**7**	F	101	181
**8**	M	108	91
**9**	M	204	15
**10**	M	121	35
**11**	F	118	51
**352**	F	71	17
**357**	F	98	164
**380**	M	180	278

### Seal-fisheries spatial overlap

**Fig 1:**
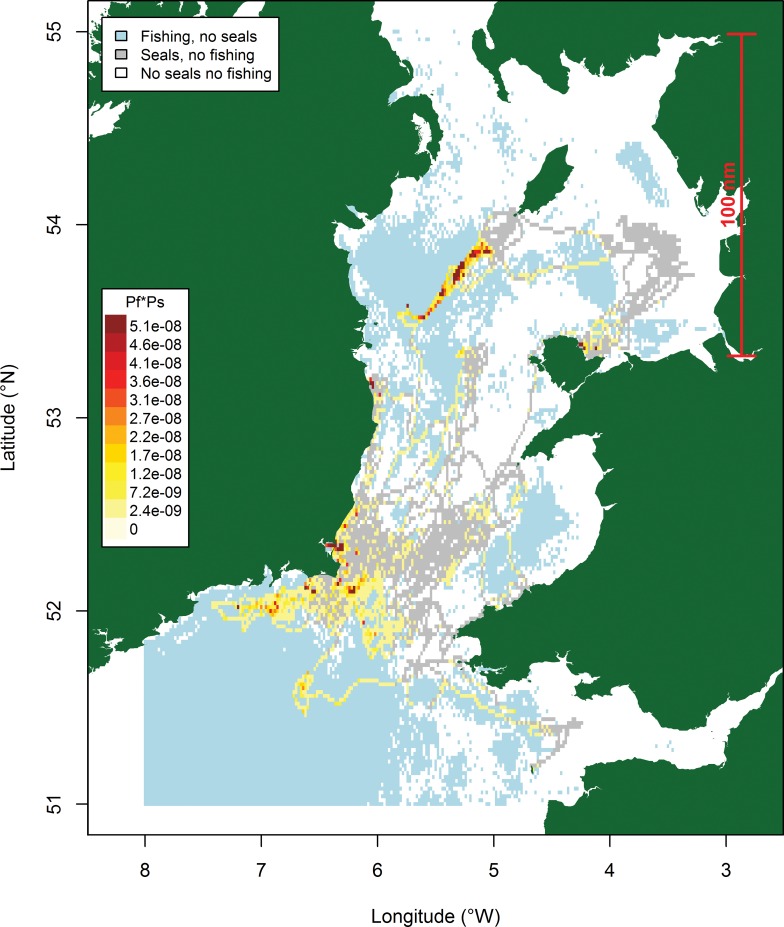
Distribution of effort of tagged grey seals, fishing vessels and areas of overlap (yellow to red) during 2013 and 2014.

**Fig 2:**
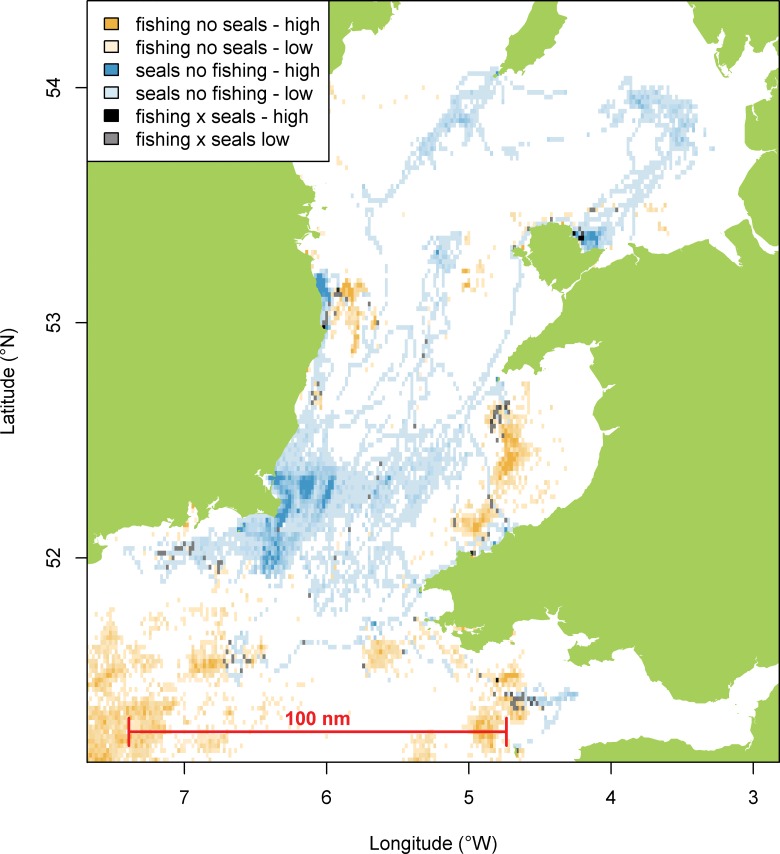
Distribution of tagged seals, passive fishing effort and areas of overlap during 2013 and 2014.

**Fig 3:**
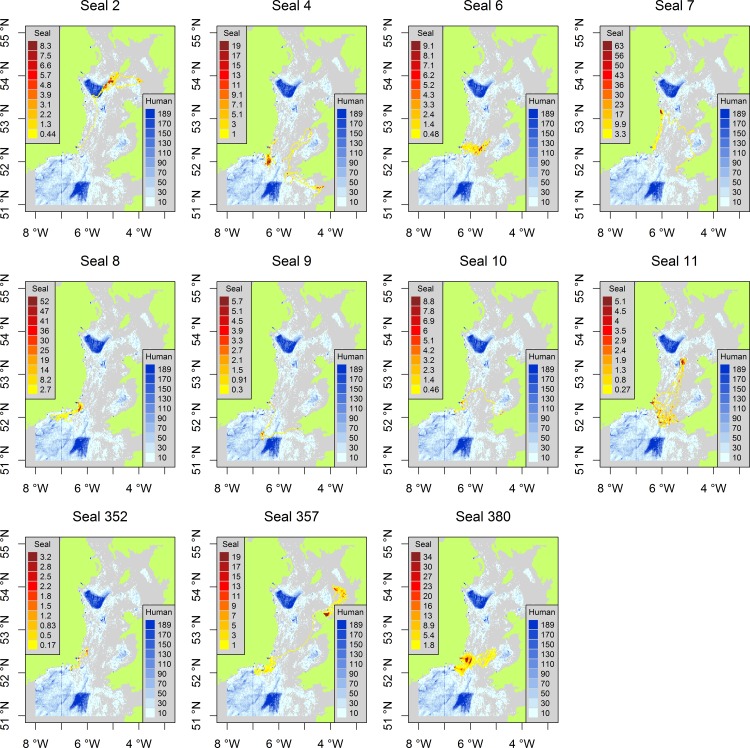
Spatial distribution of individual grey seals (yellow-red) and fishing effort (blue) of fishing vessels >12m during 2013 and 2014.

The distribution of tagged grey seals and fishing effort in the Irish and Celtic Seas clearly show different patterns in spatial distribution. This is reflected in a significantly low (p<0.001) Morista Horn Index of overlap, relative to a randomised distribution, for both active and passive fisheries (Table 2). However there are some specific areas where overlap is evident, off southeast Ireland and in the central Irish Sea, southwest of the Isle of Man (Fig 1). When we examine areas of high usage by tagged seals in more detail, we see low overlap with active fisheries but significantly high overlap with passive fisheries (p<0.01, Table 2, Fig 2). This was further examined for individual seals, which highlighted variation in seals’ patterns of overlap with fisheries (Table 2, Fig 3). Generally overlap with active fisheries was no different than one would expect from a random distribution, apart from one individual (seal 357) that showed a significantly lower overlap with these fisheries than expected by chance. Although there was an overall significant overlap between seals and passive fisheries, patterns of overlap with passive fisheries varied amongst individual seals. Five of the tagged seals showed a significantly higher than expected overlap with passive fisheries (Table 3), suggesting these seals were targeting the same foraging grounds and/or vessels/fishing gear (which includes gill nets, tangle nets, trammel nets, lines). The remaining seals displayed levels of overlap that were no different to that expected from a random distribution.

**Table 2:** Spatial overlap statistics for seals and active and passive fisheries in the Irish and Celtic Seas.

All areas	(MHI)	p value	Relative to RD*
Active gear	0.0015	**p<0.001**	**Lower**
Passive gear	0.0011	**p<0.001**	**Lower**
**Seal forage areas**			
Active gear	0.0015	p = 0.08	Lower
Passive gear	0.0391	**p<0.01**	**Higher**

*RD = a randomised distribution of seal and fishing effort. The Morisita Horn Index of overlap (MHI) varies from zero to one but in this case low levels suggest low overlap. Significance levels for C_MHf_ are provided for C*data* compared to C*random*

**Table 3:** Spatial overlap statistics for individual seals and fisheries in the Irish and Celtic Seas.

Seal	MHI Active gear		Relative to RD	MHI Passive gear		Relative to RD
**All**	0.0009	p = 0.112		0.0391	**p<0.001**	Higher
**2**	0.8870	p = 0.652		0.1010	p = 0.829	
**4**	0.0504	p = 0.534		0.0364	p = 0.194	
**6**	0.0390	p = 0.536		0.1420	**p = 0.036**	Higher
**7**	0.0265	p = 0.565		0.0102	p = 0.234	
**8**	0.0015	p = 0.730		0.0550	p = 0.331	
**9**	0.0520	p = 0.165		0.0720	**p = 0.003**	Higher
**10**	0.0119	p = 0.943		0.0035	p = 0.812	
**11**	0.0872	p = 0.803		0.0806	p = 0.082	Higher
**352**	0.0001	p = 0.627		0.1280	**p = 0.014**	Higher
**357**	0.0138	**p = 0.027**	Lower	0.0720	**p<0.001**	Higher
**380**	0.0679	p = 0.923		0.00905	**p<0.001**	Higher

RD = a randomised distribution of seal and fishing effort. The Morisita Horn Index of overlap (MHI) varies from zero to one but in this case low levels suggest low overlap. Significance levels for C_MHf_ are provided for C*data* compared to C*random*

## Discussion

The findings of this study augment previous research on grey seal and fisheries overlap in the North East Atlantic. While, much of the research on resource competition has focused on estimating biomass removal by seals and modelling the impacts of seal populations on species of high commercial value [1, 4, 22, 23], Cronin *et al*. [14] developed a novel approach that employed tracking technologies to assess spatial overlap of grey seals with fisheries. They found a significantly low rate of spatial overlap, which implied that direct competition for the resource was far less than expected. However, a number of limitations were noted, including the fact that the west coast of Ireland is dominated by a mixed whitefish and *Nephrops* trawl fishery, and the fisheries data was limited to vessels >15m length, which excludes many passive fisheries which mostly use smaller vessels. We addressed this issue in the present study by tracking grey seals that foraged in the Celtic and Irish Seas, a region containing mixed demersal and pelagic fisheries, and where data from smaller fishing vessels (12-15m) were available. Fishing effort by static or passive gears depends on a number of factors including soak time and gear dimensions, such as net length or the number of pots or hooks deployed [24], making passive gear fishing effort more difficult to quantify using VMS data than it is for towed gears [25]. Notwithstanding this, time-in-area is considered a good proxy for foraging effort in wide-ranging marine predators [20], and the removal of records where the vessels were either inactive or steaming improves our confidence that the data is representative of passive fisheries effort, as data will reflect periods of deploying and recovering gear, and it is reasonable to assume that cells with more VMS records for passive gear vessels will represent areas of higher activity. Likewise, the approach used the seals use of space as a proxy for foraging effort, enabling the quantification of spatial overlap of fisheries and seals using a consistent metric.

The present study showed variation between individuals in foraging areas. It should be noted that seals tagged at the moult site on Raven Point will include seals that breed in other areas of the grey seal range (one of our tagged seals was recorded breeding in Wales). The aim of the study was not to characterize the at-sea distribution or foraging range of post-moult seals in the Celtic and Irish Seas, but to determine whether there was evidence for interactions with fisheries when they co-occur in the same general area. Our findings largely corroborate the previous study, in that there is a low overlap between seals and fisheries using active gear. This includes demersal and pelagic trawlers despite the fact that these fisheries were targeting species such as cod, hake, haddock, Pollack, herring, and mackerel, all known to occur in the diet of grey seals in the north Atlantic [10, 11, 26]. A possible explanation is that seals may favour areas of high relief [14], which might be unsuitable for trawling and may act as refugia and contain more diverse or higher numbers of prey [27, 28]. As the current study incorporated data from smaller vessels (12-15m), and those using passive gear (e.g. gill nets, lines, tangle and trammel nets), we could examine overlap between seals and these more coastal fisheries where we believe most of the seal/fishery interactions occur [15]. Indeed overlap was evident between these passive fisheries and the tagged seals, with five of the 11 seals tagged overlapping significantly with passive fisheries, and a further seal showing high overlap which was just above significance at the p = 0.05 level. This suggests these seals may be targeting the same fishing grounds, and potentially competing (either directly through removing fish of commercial size, or indirectly by removing pre-recruits) with these fisheries. Concurrent studies, and qualitative information from the Irish fishing industry suggest seals are depredating set-nets in Irish coastal waters (and sometimes further offshore, as in the case of the hake fishery) where losses due to seal damage were estimated to be as high as 59% in certain fisheries [10, 15]. It is possible that individual seals have learnt to associate the noise of specific vessel engines and/or noise associated with hauling gear with high potential for a meal. Anecdotal evidence from fishermen supports this, as do findings from a study in the offshore deep-set hake fishery that found most damage by seals to catch occurred during net hauling [15].

Our study concurs with other assertions [10] that seal-fishery interactions in Irish waters are primarily an issue in passive (set-net) fisheries as opposed to active fisheries. It is likely that these interactions are occurring primarily at the operational level (physical interactions at nets) as opposed to competition at the biological level (removal of prey in the open sea). Diet and modelling studies in southwest Ireland suggest that seals were not likely to be competing directly with the fisheries for commercial sizes and species of fish in the biologically sensitive area [10, 13]. Net-foraging may be a learnt behaviour, reinforced with the reward of an ‘easy meal’ that requires minimal energy to acquire. Studies in the North and Baltic Seas suggest that depredation in salmon rivers and at fish traps was caused by a relatively small number of ‘specialised’ individual grey seals who repetitively returned to depredate [29, 30]. Similar behavioural traits have been observed in terrestrial carnivores, with ‘problem individuals’ assumed to be responsible for most cases of livestock depredation [31]. This suggests that predator control measures should be specifically targeted at problem individuals, as opposed to an indiscriminate cull or population reduction. Removal of individuals by capture and/or shooting is practical only in a limited number of fisheries e.g. pontoon salmon traps [30] and more often than not depredation by seals occurs out of sight of fishermen. Other practical mitigation measures to reduce depredation (and seal by-catch) in these fisheries should be explored fully, including the potential use of acoustic startle devices [32, 33].

Our study highlights the importance of differentiating between fisheries in analyses of top predator/fishery interactions, as overlap was noted with passive rather than active fisheries operating in the same general area. Ours is not the first study to identify fishery-specific top predator behaviour; Bodey *et al*. [34] demonstrated that tagged gannets were more likely to switch to foraging behaviour around trawlers than non-trawlers, particularly when the vessel behaviour indicated that discards were more likely to be available; killer whales and sperm whales have learned to target specific commercial long-line fisheries [35, 36]; and bottlenose dolphins have been observed to specifically target net fisheries for shrimp [37].

A limitation of the current study in terms of using the telemetry data to identify fine-scale behaviour is the infrequency of at-sea GPS locations. Compared to other tracking studies of marine predators such as seabirds where locations can be as frequent as every minute, seals spend a much higher proportion of time underwater, and despite programming tags to acquire fixes every 30 minutes, they are only able to do so when the seal is at the surface. This has resulted in an average daily number of at-sea locations of 12. While this is consistent with the frequency of VMS data, this could be increased by re-programming tags to capture data more frequently and including accelerometers to gain information on fine-scale behaviour. However, this comes at a cost to battery life, and additional costs associated with transmission of location and dive data via the GSM mobile phone network. Nevertheless, a shorter but more data-rich tagging period would provide a means to examine the track data in more detail. In-depth analyses of dive shape [38] could be used to more accurately identify and differentiate foraging dives, search dives, travel, and rest, and techniques such as Markov chain analyses [34] could then be used to investigate how seals react to vessel proximity at a much finer scale than was possible using the infrequent GPS fixes obtained in this study.

Quantifying the scale of seal-fishery interaction remains a challenge. Whilst observer programs provide valuable data on depredation, they are expensive, time-consuming and generally limited to a small number of vessels. Furthermore, the likelihood of interactions (including damage to catch and gear) is likely to vary according to a number of factors, not least of all the proximity of fishery to seal colonies/haul-out sites. Studies such as this augment existing observer programs by providing an overview of spatial overlap and a mechanism to identify areas of high overlap for targeted studies. Modelling the foraging distribution of tagged seals [39] with vessel distribution (from VMS data) and taking into account fishery specific depredation and by-catch levels (from observer programs) will be a useful means of predicting areas and fisheries most at risk of seal depredation and by-catch. Such information will not only inform conservation status assessment (required for Annex II species under the EU Habitats Directive), it would contribute to the Marine Strategy Framework Directive (levels of seal by-catch as a common indicator of Good Environmental Status) and will be useful to resource managers in developing fisheries management plans under an ecosystems approach to fisheries management.

## Supporting Information

S1 Appendix Active and passive gear vms and seal effort data_seal forage areas. (XLSX)

## References

[1] Bundy A, Heymans JJ, Morissette L, Savenkoff C. Seals, cod and forage fish: A comparative exploration of variations in the theme of stock collapse and ecosystem change in four Northwest Atlantic ecosystems. Progress In Oceanography. 2009;81:188–206.

[2] Pope JG, Holmes SJ. Length-based approaches compared to age-based approaches to determining the significance of grey seal feeding on cod in ICES Division VIa. ICES CM. 2008;/F:08.

[3] Trzcinski MK, Mohn R, Bowen WD. Continued decline of an Atlantic cod population: how important is gray seal predation? Ecological Applications. 2006;16(6):2276–92. 17205904 10.1890/1051-0761(2006)016[2276:cdoaac]2.0.co;2

[4] Cook RM, Holmes SJ, Fryer RJ. Grey seal predation impairs recovery of an over‐exploited fish stock. Journal of Applied Ecology. 2015;52(4):969–79.

[5] Tudela S. Ecosystem effects of fishing in the Mediterranean: an analysis of the major threats. GFCM Stud Rev. 2004;74:1–44.

[6] Varjopuro R. Co-existence of seals and fisheries? Adaptation of a coastal fishery for recovery of the Baltic grey seal. Marine Policy. 2011;35(4):450–6.

[7] Enstipp MR, Daunt F, Wanless S, Humphreys EM, Hamer KC, Benvenuti S, et al Foraging energetics of North Sea birds confronted with fluctuating prey availability In: Boyd IL, Wanless S, Camphuysen CJ, editors. Top predators in marine ecosystems: their role in monitoring and management. Cambridge: Cambridge University Press; 2006.

[8] Hui TC, Gryba R, Gregr EJ, Trites AW. Assessment of Competition between Fisheries and Steller Sea Lions in Alaska Based on Estimated Prey Biomass, Fisheries Removals and Predator Foraging Behaviour. PloS one. 2015;10(5):e0123786 10.1371/journal.pone.0123786 25950178 PMC4424003

[9] Punt A, Butterworth D. The effects of future consumption by the Cape fur seal on catches and catch rates of the Cape hakes. 4. Modelling the biological interaction between Cape fur seals Arctocephalus pusillus pusillus and the Cape hakes Merluccius capensis and M. paradoxus. South African Journal of Marine Science. 1995;16(1):255–85.

[10] Cronin M, Gosch M, Jessopp M, Luck C, Rogan E, Reid D. A Pilot Study of Seal Predation on Salmon Stocks in Selected Irish Rivers and Estuaries. Final report to IFI. Coastal & Marine Research Centre, University College Cork, 2014.

[11] Bowen W, Lawson J, Beck B. Seasonal and geographic variation in the species composition and size of prey consumed by grey seals (Halichoerus grypus) on the Scotian Shelf. Canadian Journal of Fisheries and Aquatic Sciences. 1993;50(8):1768–78.

[12] Brown EG, Pierce GJ. Monthly variation in the diet of harbour seals in inshore waters along the southeast Shetland (UK) coastline. Marine Ecology Progress Series. 1998;167:275–89.

[13] Houle JE, Castro F, Cronin MA, Farnsworth KD, Gosch M, Reid DG. Effects of seal predation on a modelled marine fish community and consequences for a commercial fishery. Journal of Applied Ecology. 2016;53(1):54–63.

[14] Anderwald P, Haberlin MD, Coleman M, Ó Cadhla O, Englund A, Visser F, et al Seasonal trends and spatial differences in marine mammal occurrence in Broadhaven Bay, north-west Ireland. Journal of the Marine Biological Association of the United Kingdom. 2012;1(1):1–10.

[15] Cosgrove R, Gosch M, Reid D, Sheridan M, Chopin N, Jessopp M, et al Seal depredation in bottom-set gillnet and entangling net fisheries in Irish waters. Fisheries Research. 2015;172:335–44.

[16] Cosgrove R, Gosch M, Reid D, Sheridan M, Chopin N, Jessopp M, et al Seal bycatch in gillnet and entangling net fisheries in Irish waters. Fisheries Research. 2016;183:192–9. 10.1016/j.fishres.2016.06.007

[17] Cosgrove R, Cronin M, Reid D, Gosch M, Sheridan M, Chopin N, et al Seal depredation and bycatch in set net fisheries in Irish waters. Fisheries Resource Series Vol 10 Dublin: 2013.

[18] Cronin M, Kavanagh A, Rogan E. The foraging ecology of the harbour seal (Phoca vitulina vitulina) in southwest Ireland. Final report to the Marine Institute St/05/12. 145 pp., 2008.

[19] Gerritsen H, Lordan. Integrating VMS data with catch data from logbooks. 2010.

[20] Warwick-Evans V, Atkinson PW, Gauvain RD, Robinson LA Arnould JPY, Green JA. Time-in-area represents foraging activity in a wide-ranging pelagic forager. Marine Ecology Progress Series. 2015;527:233–46.

[21] Corkeron P. Marine mammals' influence on ecosystem processes affecting fisheries in the Barents Sea is trivial. Biology letters. 2009;5:204–6. 10.1098/rsbl.2008.0628 19126534 PMC2665811

[22] Butler JRA, Middlemas SJ, Graham IM, Thompson PM, Armstrong JD. Modelling the impacts of removing seal predation from Atlantic salmon, Salmo salar, rivers in Scotland: a tool for targeting conflict resolution. Fisheries Management and Ecology. 2006;13:285–91. 10.1111/j.1365-2400.2006.00504.x

[23] Hammill M, Stenson G, Swain D, Benoît H. Feeding by grey seals on endangered stocks of Atlantic cod and white hake. ICES Journal of Marine Science: Journal du Conseil. 2014:fsu123.

[24] Waugh SM, Filippi DP, Kirby DS, Abraham E, Walker N. Ecological Risk Assessment for seabird interactions in Western and Central Pacific longline fisheries. Marine Policy. 2012;36(4):933–46.

[25] Lee J, South aB, Jennings S. Developing reliable, repeatable, and accessible methods to provide high-resolution estimates of fishing-effort distributions from vessel monitoring system (VMS) data. ICES Journal of Marine Science. 2010;67:1260–71.

[26] Hammond P, Hall A, Prime J. The diet of grey seals around Orkney and other island and mainland sites in north-eastern Scotland. Journal of Applied Ecology. 1994:340–50.

[27] Jaworski A, Solmundsson J, Ragnarsson SA. The effect of area closures on the demersal fish community off the east coast of Iceland. ICES Journal of Marine Science: Journal du Conseil. 2006;63(5):897–911.

[28] Busch M, Kannen A, Garthe S, Jessopp M. Consequences of a cumulative perspective on marine environmental impacts: offshore wind farming and seabirds at North Sea scale in context of the EU Marine Strategy Framework Directive. Ocean & Coastal Management. 2013;71:213–24. 10.1016/j.ocecoaman.2012.10.016

[29] Butler JR, Middlemass S, Graham I, Harris R. Perceptions and costs of seal impacts on Atlantic salmon fisheries in the Moray Firth Scotland: Implications fr the adaptive co-management of seal-fishery conflict. Marine Policy. 2011;3:317–23.

[30] Königson S, Fjälling A, Berglind M, Lunneryd S-G. Male gray seals specialize in raiding salmon traps. Fisheries Research. 2013;148:117–23.

[31] Linnell JCD, J., Odden J, Smith ME, Aanes R, Swenson JE. Large carnivores that kill livestock: do" problem individuals" really exist? Wildlife Society Bulletin. 1999;27:698–705.

[32] Götz T, Janik V. Non‐lethal management of carnivore predation: long‐term tests with a startle reflex‐based deterrence system on a fish farm. Animal Conservation. 2016.

[33] Götz T, Janik VM. Acoustic deterrent devices to prevent pinniped depredation: efficiency, conservation concerns and possible solutions. Marine Ecology Progress Series. 2013;492:285–302.

[34] Bodey TW, Jessopp MJ, Votier SC, Gerritsen HD, Cleasby IR, Hamer KC, et al Seabird movement reveals the ecological footprint of fishing vessels. Current Biology. 2014;24(11):R514–R5. 10.1016/j.cub.2014.04.041 24892908

[35] Peterson MJ, Carothers C. Whale interactions with Alaskan sablefish and Pacific halibut fisheries: surveying fishermen perception, changing fishing practices and mitigation. Marine Policy. 2013;42:315–24.

[36] Visser IN. Killer whale (Orcinus orca) interactions with longline fisheries in New Zealand waters. Aquatic mammals. 2000;26(3):241–52.

[37] Leatherwood S. Some observations of feeding behavior of bottle-nosed dolphins (Tursiops truncatus) in the northern Gulf of Mexico and (Tursiops cf. T. gilli) off southern California, Baja California, and Nayarit, Mexico. Marine Fisheries Review. 1975;37(9):10–6.

[38] Baechler J, Beck C. Dive shapes reveal temporal changes in the foraging behaviour of different age and sex classes of harbour seals (Phoca vitulina). Canadian journal of. 2002;1577:1569–77. 10.1139/Z02-150

[39] Jones EL, McConnell BJ, Smout S, Hammond PS, Duck CD, Morris CD, et al Patterns of space use in sympatric marine colonial predators reveal scales of spatial partitioning. Marine Ecology Progress Series. 2015;534:235–49.

